# Supplementary material for: Effects of Sucrose Feeding on the Quality of Royal Jelly Produced by Honeybee Apis mellifera L
Source: Insects. 2023 Sep 4;14(9):742. doi: 10.3390/insects14090742 (PMC10532100; doi:10.3390/insects14090742)
Supplement: Supplementary file 1 [file insects-14-00742-s001.zip › Figure S1.pdf]

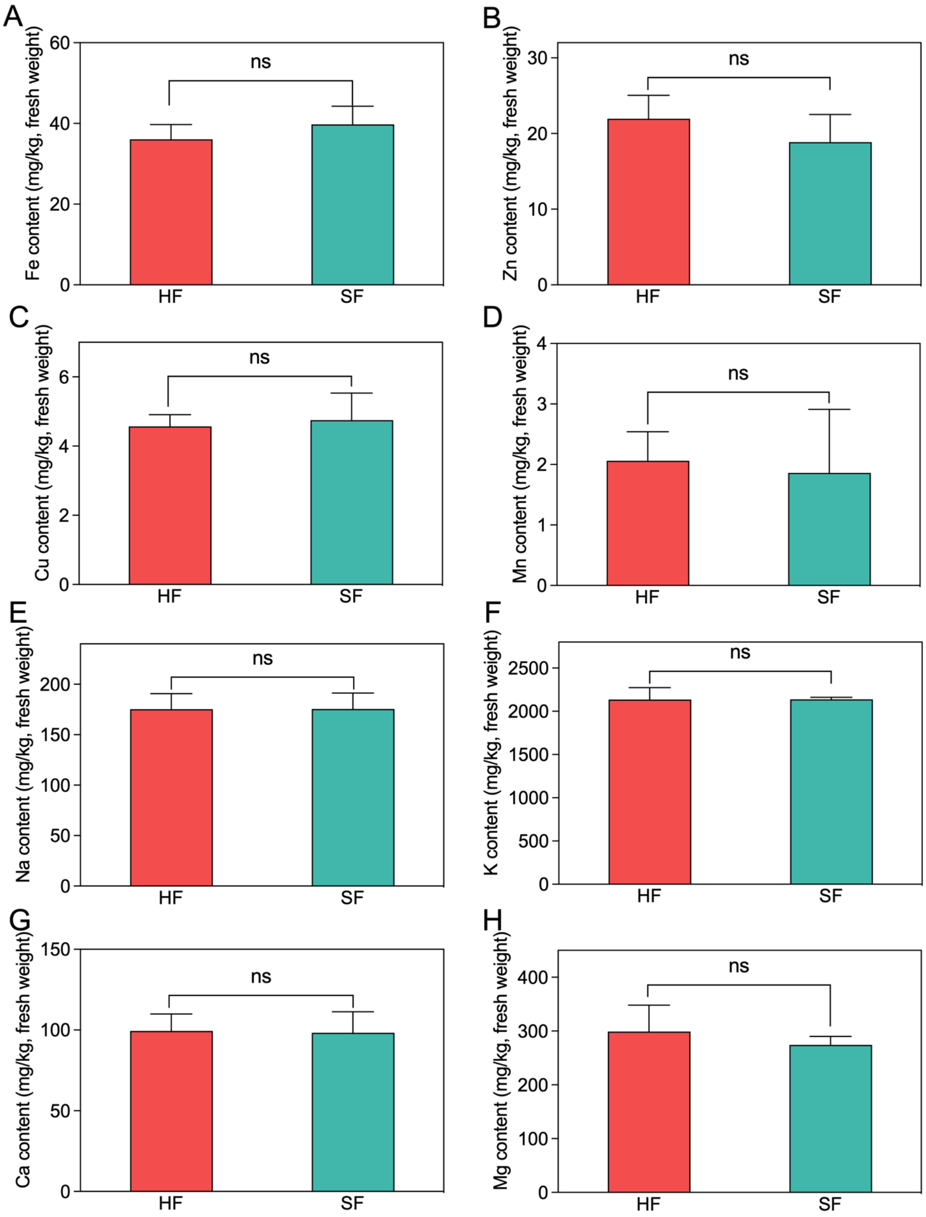

Figure S1. Mineral element content of analyzed RJ samples derived from honey feeding and sucrose feeding procedures (mg/kg, fresh weight). HF, honey feeding group; and SF, sucrose feeding group. Values are means ± SD. ** $P < 0.01$ by independent samples t-test.
