# Supplementary material for: Effects of Sucrose Feeding on the Quality of Royal Jelly Produced by Honeybee Apis mellifera L
Source: Insects. 2023 Sep 4;14(9):742. doi: 10.3390/insects14090742 (PMC10532100; doi:10.3390/insects14090742)
Supplement: Supplementary file 1 [file insects-14-00742-s001.zip › Figure S2.pdf]

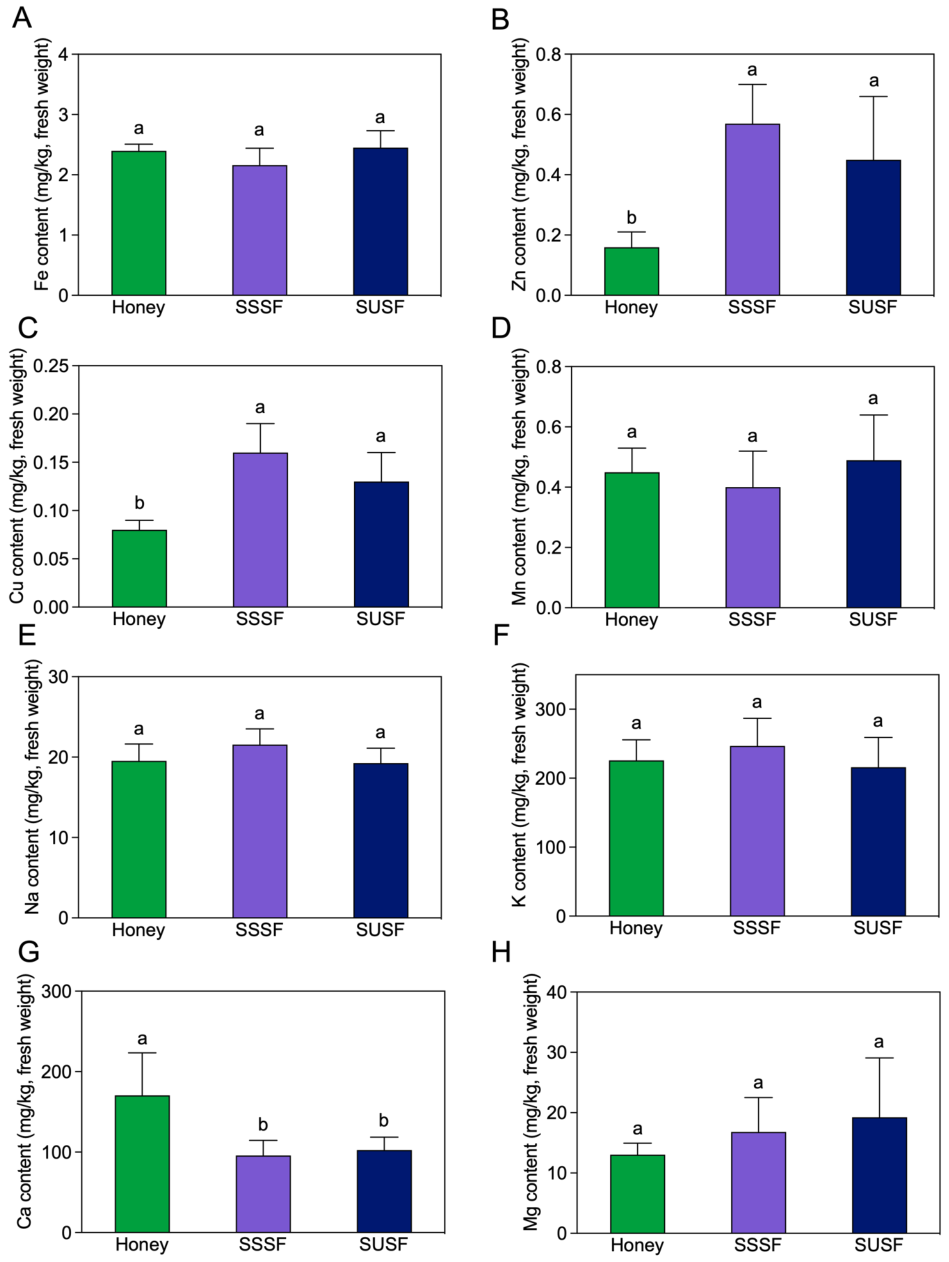

Figure S2. Comparison of element contents of stored food obtained from honey-fed and sucrose-fed colonies (mg/kg, fresh weight). Honey, stored food obtained from honey-fed colonies; SSSF, stored food obtained from sealed combs of sucrose-fed colonies; and SUSF, stored food obtained from unsealed combs of sucrose-fed colonies. Values are means ± SD. Different lowercase letters indicate significant differences among treatments at the 0.05 level, according to the Tukey's HSD test.
