# Supplementary material for: Effects of Sucrose Feeding on the Quality of Royal Jelly Produced by Honeybee Apis mellifera L
Source: Insects. 2023 Sep 4;14(9):742. doi: 10.3390/insects14090742 (PMC10532100; doi:10.3390/insects14090742)
Supplement: Supplementary file 1 [file insects-14-00742-s001.zip › Table S1.pdf]

Table S1. Information on the primers used for qRT-PCR in this experiment.

Genes	GenBank accession number	Primer sequence (5' - 3')	Source
MRJP1	GB55205	CACAGCCCAAGATGGAATTT	Wu et al., 2017 [39]
		AAGAGGACGCCACTCTTTGA	
MRJP3	GB55204	ATTGCCGTAAACGCCACTAC	
		CAATCGATGGAAGGAATCGT	
